# An Adaptive Motion Estimation Scheme for Video Coding

**DOI:** 10.1155/2014/381056

**Published:** 2014-02-11

**Authors:** Pengyu Liu, Yuan Gao, Kebin Jia

**Affiliations:** School of Electronic Information & Control Engineering, Beijing University of Technology, Beijing 100124, China

## Abstract

The unsymmetrical-cross multihexagon-grid search (UMHexagonS) is one of the best fast Motion Estimation (ME) algorithms in video encoding software. It achieves an excellent coding performance by using hybrid block matching search pattern and multiple initial search point predictors at the cost of the computational complexity of ME increased. Reducing time consuming of ME is one of the key factors to improve video coding efficiency. In this paper, we propose an adaptive motion estimation scheme to further reduce the calculation redundancy of UMHexagonS. Firstly, new motion estimation search patterns have been designed according to the statistical results of motion vector (MV) distribution information. Then, design a MV distribution prediction method, including prediction of the size of MV and the direction of MV. At last, according to the MV distribution prediction results, achieve self-adaptive subregional searching by the new estimation search patterns. Experimental results show that more than 50% of total search points are dramatically reduced compared to the UMHexagonS algorithm in JM 18.4 of H.264/AVC. As a result, the proposed algorithm scheme can save the ME time up to 20.86% while the rate-distortion performance is not compromised.

## 1. Introduction

Advances in the mobile communication technologies have enabled portable devices to run complex multimedia applications involving video processing. Due to the rapid growth of the multimedia service, the video compression becomes essential for reducing the required bandwidth for transmission and storage in many applications. In video compression, motion estimation (ME) is the most crucial part since it can reduce the total video data efficiently by exploiting the temporal correlation among successive frames of a video sequence to achieve a high data compression ratio.

The block-matching algorithm (BMA) based ME has been widely used in many video compression standards, such as the H.26x and the MPEG-x families because of its simplicity and effectiveness. The previous video coding standards (H.261, H.263, etc.) adopt the fixed block size motion estimation, which uses the same block size for both static and moving objects. The latest H.264/AVC [1] provides better estimation of small and irregular motion fields in a video sequence by supporting variable block size ME, which segments each MB into seven types of subblocks (4 × 4, 4 × 8, 8 × 4, 8 × 8, 8 × 16, 16 × 8, and 16 × 16).

Although H.264/AVC achieves significantly better coding performance compared to previous video coding standards, the coding complexity is more complicated, since calculations over the seven block types are needed to generate the motion vector (MV). In [2, 3], it has been proved that the ME process contributes the majority of the computational complexity; ME process can consume 70% (one reference frame) to 90% (five reference frames) of total encoding time of a standard H.264/AVC encoder. Therefore, H.264/AVC and even HEVC (high efficiency video coding) are all dedicated to the study of efficient motion estimation algorithm in order to reduce the encoding time and improve the encoding efficiency.

In recent years, many fast ME algorithms have been proposed to accelerate the ME operation, such as three step search (TSS) [4], four step search (FSS) [5], diamond search (DS) [6], and hexagon search (HS) [7]. These ME algorithms typically consist of two steps: one is prediction process and the other is block-matching search process.

In prediction process, more predictors are utilized to locate the initial search point, such as median predictor, up-layer predictor, corresponding-block predictor, and neighboring ref-frame predictor [8]. In addition, in order to early stop block-matching process, the prediction process is also applied to compute the early termination thresholds. In block-matching process, in order to find the best matching block by employing one or more block-matching search patterns, such as square search pattern, cross search pattern, hexagon search pattern, and diamond search pattern.

These fast algorithms are all based on the assumption that the block-matching error is monotonic decrease in the search window. However, this assumption may result in a local minimum.

In order to avoid the local minimum problem, some self-adaptive search algorithms are proposed, such as predictive MV field adaptive search technique (PMVFAST) algorithm [9] and enhanced predictive zonal search (EPZS) algorithm [10]. To some degree, these classical motion estimation search algorithms are regarded as the foundations of future researches.

Up to now, a lot of advanced motion estimation search algorithms have been proposed. Song and Akoglu [11] raised variable block size motion estimation architecture and optimized in hardware. Park [12] developed a search strategy based on multiple reference frames. Choi and Jeong [13] proposed a constrained two-bit transform for low complexity motion estimation. Kim et al. [14] proposed a novel motion estimation algorithm based on spectral image analysis and statistical object. Chen et al. [15] firstly defined the clustering feature of MVs and reduction of the motion estimation time successfully. The above novel motion estimation algorithms not only make full use of the advantages of H.264/AVC, but also combine the statistics with the scheme.

With the further study of the temporal and spatial correlation and human visual characteristics, the new algorithm has made a progress. The typical algorithm is unsymmetrical-cross multihexagon search (UMHexagonS) algorithm. It employs more than one predictor to locate the initial search point and then utilizes hybrid block-matching search pattern to find the best matching block. In particular, in order to further reduce the computational complexity of block-matching process, they utilize an adaptive early termination strategy to early stop the block-matching process.

UMHexagonS algorithm develops reasonable search patterns. Among these fast ME algorithms, UMHexagonS is successful in the fact that the majority ME time is saved from the full search (FS) while the R-D performance is not compromised, it has been adopted in H.264/AVC reference software JM7.6 [16]. However, the outstanding coding performance of UMHexagonS is at the cost of relatively high computational complexity of the hybrid block-matching search pattern. Hence, if the block-matching search patterns of UMHexagonS are simplified, much more ME time will be saved.

Aiming at calculation redundancy, some proposed algorithms based on UMHexagonS have been improved. Li and Yang [17] developed some new techniques to improve the UMHexagonS algorithm, such as dynamic search window, motion type adaptive search strategies, directional cross-shaped search, adaptive rectangle-diamond search, adaptive multilevels octagon regional search, and adaptive hexagon search. Yang et al. [18] proposed a new early termination threshold and partitioned search patterns. Wu et al. [19] developed dynamic search range selection, big hexagon, and small hexagon search mode. Chen et al. [20] optimized the integer pixel search algorithm and improved a subpixel search based on UMHexagonS algorithm. For the application of ME algorithm in hardware, Lifen et al. [21] reduced computational complexity by using few search points without degrading image quality and applied the modified patterns with new uneven cross, multihexagon grid and hexagon. Huayi et al. [22] combined the single instruction multiple data with software algorithm. Jambek et al. [23] set up the architecture that consists of pixel buffers, processing elements, adder tree, comparator unit, and control unit based on UMHexagonS algorithm. Besides, New-UMHexagonS (NUMHexagonS) algorithm involves the preliminary discussions on macroblock correlation [24]. The NUMHexagonS algorithm achieved good effect on optimizing UMHexagonS algorithm. In spite of the fact that the above algorithms can reduce motion estimation time in varying degrees, mining and use of motion characteristics in macroblock still need further research.

This paper proposes a novel motion estimation search algorithm. It makes full use of MV distribution characteristics to narrow the search range and then designs new search patterns based on motion features, at last achieves selecting search areas adaptively. The proposed algorithm enhances the performance of UMHexagonS algorithm remarkably on the condition that it maintains a low bit rate and high video quality.

The remainder of this paper is organized as follows. In Section 2, some related works about the motion estimation, UMHexagonS algorithm, and NUMHexagonS algorithm are introduced as the fundamental research.  Section 3 analyses the MV distribution characteristics during the ME process.  Section 4 describes a method of predicting MV distribution and a search strategy with new search patterns. Experimental results are given to verify the effectiveness of the proposed algorithm in Section 5.  Section 6 draws the conclusions.

## 2. Related Works

**Figure 1:**
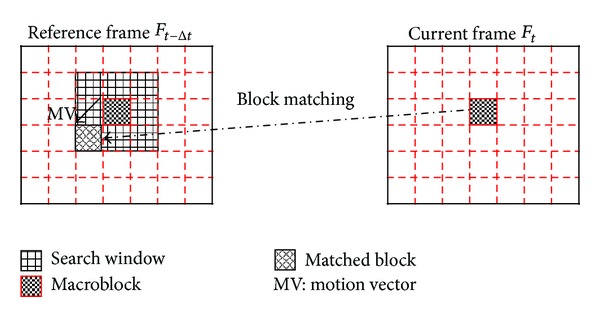
Block-matching process.

H.264/AVC accepts block-matching technique for motion estimation. Block-matching technique partitions the current frame into many macroblocks, such as 16 × 16, 8 × 16, 16 × 8, 8 × 8, 8 × 4, 4 × 8, and 4 × 4. Each macroblock performs motion estimation and regards computational result as a candidate MV.  Figure 1 shows the block-matching process in H.264/AVC. The current frame and the reference frame are divided into macroblocks and each block is matched at all locations within the search window of the previous frame.

In integer prediction for most of the motion estimation search algorithms, the criterion to obtain MV is the sum of absolute differences (SAD). SAD is defined as follows:

(1) SAD[s,r(ref,m)] =∑x=1,y=1M,N|s[x,y]−r[x−mx,y−my]|.

*M*  and *N* are the width and height of the current macroblock, respectively; *x* and *y* is the coordinate of the current macroblock; *s* = *s*[*x*, *y*] is the actual value; *r* is the predicted value which is depend on ref and *m*; ref is the value of the reference frame; *m* = [*m*_*x*_, *m*_*y*_] is the coordinate of the current MV; *m*_*x*_ and *m*_*y*_ are the motion components in horizontal and vertical, respectively.

During motion estimation search, the minimum SAD is chosen and the corresponding MV is regarded as a candidate MV. Because of the large computational working, motion estimation search costs the main time of the whole encoding process.

Compared to full search algorithm, UMHexagonS algorithm claims that it can reduce 90% of motion estimation time, drop less than 0.05 dB PSNR, and maintain a low bit rate. In order to make the initial search point close to the best prediction point, UMHexagonS algorithm provides several different initial search point predictions. UMHexagonS algorithm searching strategy begins with cursory search pattern, then turns to elaborate search patterns. With multipatterns, UMHexagonS algorithm gets rid of the disadvantage that the traditional fast algorithms are easy to trap in local minima. In addition, the self-adaptive early termination threshold makes UMHexagonS algorithm more efficient by cutting out the searching process. To sum up, UMHexagonS algorithm improves the effectiveness and robustness of the prediction greatly.

Apart from the initial search point prediction, it causes a lot of unnecessary search points during the search process that UMHexagonS algorithm does not combine pattern search with MV characteristics. Because of the nonuniform distributed MVs in each search step, there is no need to traverse all search points to determine the best matched point. The large search pattern does not take motion characteristics into consideration and point-by-point blind searching contributes little to improving the accuracy of motion estimation, while consuming lots of encoding time. On the basis of the above features, in previous work, we did some researches and proposed NUMHexagonS algorithm. NUMHexagonS algorithm improves the following ways to optimize:

1. based on the feature that layers of search points are progressively decreasing by search radius decreasing designs a new uneven multihexagon-grid search pattern;
2. based on the macroblock motion intensity adaptively selects the layers of the uneven multihexagon-grid search pattern;
3. based on the macroblock motion intensity adaptively selects whether perform the 5 × 5 full search pattern.

**Figure 2:**
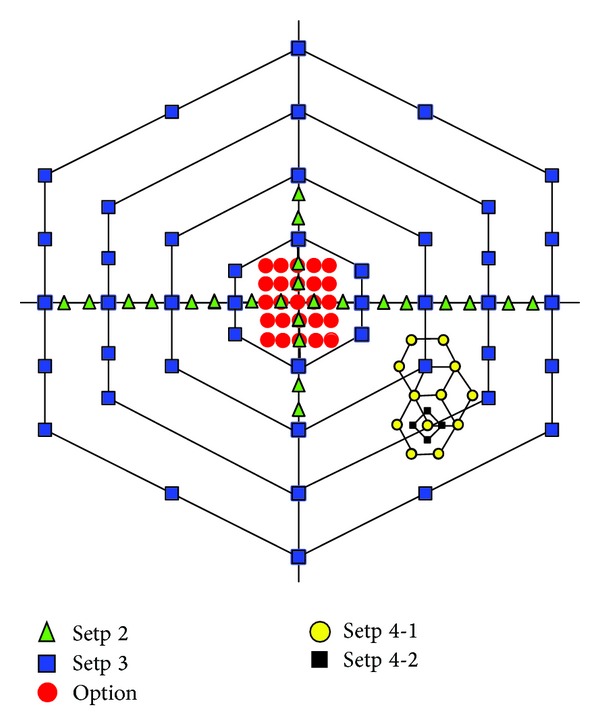
Search process and search pattern of NUMHexagonS.

NUMHexagonS algorithm search process is shown in Figure 2. Compared to UMHexagonS algorithm, the PSNR is almost the same, while increasing 0.24% bit rate on average and reducing 23% motion estimation time on average.

Although NUMHexagonS algorithm makes a good progress in reducing motion estimation time, it is still rough that NUMHexagonS algorithm combines MV characteristics with search strategy. It is not precise to predict the MV that NUMHexagonS algorithm only carries out the macroblock motion intensity. Intensity is one of the MV characteristics, but it lacks direction information. This paper will make better use of MV characteristics based on NUMHexagonS algorithm. The statistic of MVs distribution will be figured out and depend on that we develop a more precise search strategy. Next section will mainly analyse MV distribution characteristics.

## 3. MV Distribution Statistics

The uneven multihexagon-grid search used in UMHexagonS algorithm has a wide search range and costs lots of search points; hence, it takes too much time. Therefore, it is very necessary to research the feature of MV distribution.

**Figure 3:**
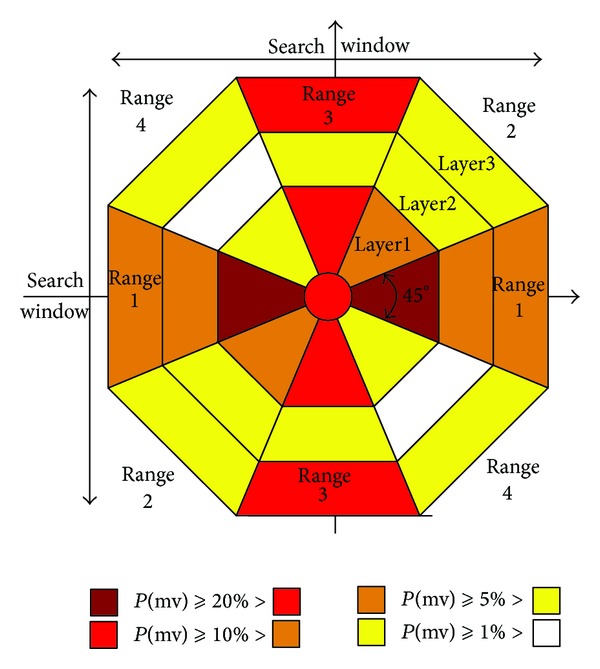
MV distribution probability.

In order to obtain the statistic of MV distribution, the search window is divided into multilayer octagon as shown in Figure 3.

Central region indicates no motion, and it means that the MV is similar to 0. Other regions indicate the positions where the best matched points appear. Thus, all regions are defined as follows:

(2) central  regionOrigin(−22.5°,22.5°]∪(157.5°,202.5°]Range1(22.5°,67.5°]∪(−157.5°,−112.5°]Range2(67.5°,112.5°]∪(−112.5°,−67.5°]Range3(112.5°,157.5°]∪(−67.5°,−22.5°]Range4

In (2) each Range is divided into Layer1, Layer2, and Layer3 from inside to outside and thereby constitutes 13 regions. Take H.264/AVC software JM18.4 as experimental platform and select seven random QCIF format (176 × 144) standard test sequences to figure out the best matched point probability that appears in each region of uneven multihexagon-grid search. The statistical results are shown in Table 1.

**Figure 4:**
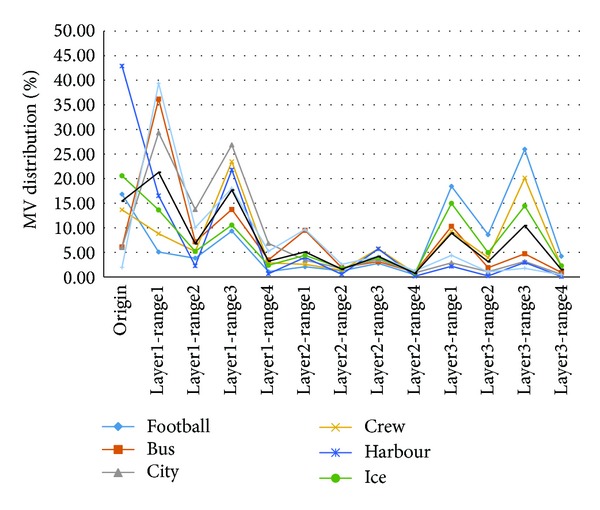
MV distribution curves of each sequence.

According to Table 1, the curves of MV distribution are drawn in Figure 4. For the video sequence HARBOUR with low motion, the best matched points mainly appear in Origin (42.9%) and Layer1-Range3 (21.78%), which indicates that the majority of macroblock MVs concentrate in central region. For the video sequence FOOTBALL with high motion, the best matched points mainly appear in Layer3-Range1 (18.44%) and Layer3-Range3 (25.96%), which indicates that the majority of macroblock MVs concentrate on boundary. For video sequence BUS whose foreground motion is low and background motion is high, the best matched points appear intensively in Layer1-Range1 (36.18%), Layer1-Range3 (13.74%), and Layer3-Range1 (10.31%), which indicates that distribution of the macroblock MVs is dispersed. The Total Average curve shows that the mean MVs of the seven sequences distribute in Origin (15.44%), Layer1-Range1 (21.28%), Layer1-Range3 (17.69%), Layer3-Range1 (8.92%), and Layer3-Range3 (10.45%) unevenly. The above analyses show that there is an intrinsic link between the MV distribution and the motion estimation search position.

In order to further investigate the characteristics of MV distribution, figure out the average numbers which are every sequence in the same region in Table 1 and then use different colors for plotting in Figure 3. The colors that varied from dark to light represent MV distribution probabilities varied from higher to lower. The statistical results show that MV distribution meets the following characteristics.

1. Motion vectors located in horizontal and vertical are more than that in other directions and the distribution probability in horizontal is higher than that in vertical.
2. A large number of MV's intensity is close to zero.
3. Near the center or the boundary of search window appears higher probability of MVs.

Characteristic (1) complies with the phenomena that most of video sequence motions exist in horizontal more than in vertical, like human walking, boating, car running, and so forth. Characteristic (2) indicates that the video sequence with low-motion macroblocks shows a high probability of MV distribution in the origin or near the origin, like broadcasting, rotating shoot, and so forth, and also including most of static backgrounds. Characteristic (3) indicates that the video sequence with high motion macroblock appears a high probability of MV distribution at the boundary, like the object moving out of the search window. According to the above characteristics, the search strategy cannot achieve the purpose of accurate search which only distinguishes video sequences simply by low motion or high motion. Therefore, it is necessary to refine those search patterns based on MV distribution, so that it can make motion estimation process more accurately and reduce search points further to raise search efficiency.

## 4. Principle of the Proposed ME Scheme

Based on MV distribution characteristics, the proposed algorithm makes NUMHexagonS algorithm further optimized. The patterns of NUMHexagonS algorithm are divided into different areas which are selected adaptively by MV distribution prediction, thereby the proposed algorithm achieves reducing the motion estimation search points.

### 4.1. Design Patterns

Unsymmetrical cross search and uneven multihexagon-grid search belong to cursory search processes with wide search ranges. The MV distribution prediction can pinpoint the search position without large search range, so it is necessary to divide the original patterns into different areas and draw up new search strategies.

**Figure 5:**
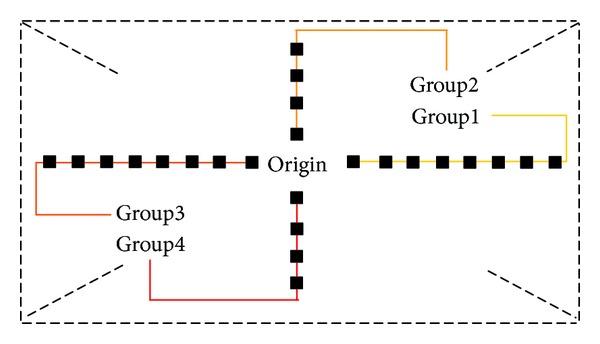
Modified unsymmetrical cross search pattern.

The unsymmetrical cross search pattern of NUMHexagonS algorithm is divided into four Groups as shown in Figure 5. There are 8 search points in horizontal and 4 search points in vertical, respectively. The modified unsymmetrical cross search pattern complies with the requirement of characteristic (1) mentioned in Section 3. During the motion estimation, the matched point is searched in one of the four Groups determined by MV distribution prediction. The search points of the modified unsymmetrical cross search pattern are compressed to 1/3 (in Group1 or Group3) or 1/6 (in Group2 or Group4) compared to the original pattern.

**Figure 6:**
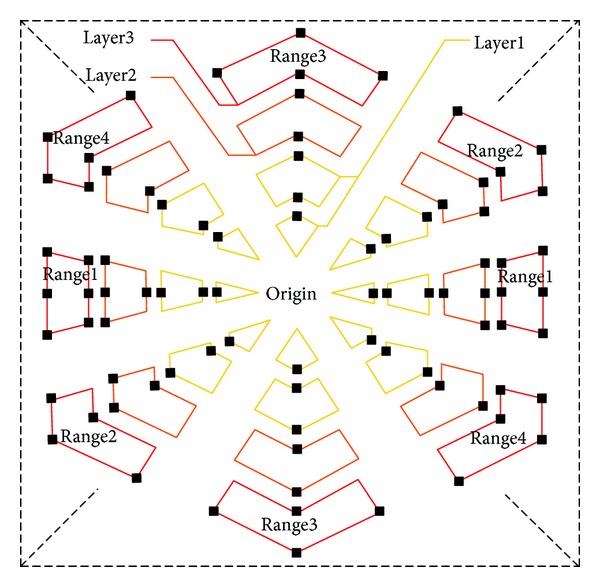
Modified uneven multihexagon-grid search pattern.

The uneven multihexagon-grid search pattern of NUMHexagonS algorithm is divided into 32 regions as shown in Figure 6. To comply with the requirement of characteristic (3) mentioned in Section 3, the search points are distributed unevenly. More search points are distributed closer to the center and the boundary. Layer1 contains 16 regions total 24 search points. Layer2 contains 4 regions total 22 search points. Layer3 contains 4 regions total 38 search points. In order to comply with the requirement of characteristic (1) mentioned in Section 3, there are 62 total search points distributed in horizontal ±45° direction and 22 total search points distributed in vertical ±45° direction. During the motion estimation the matched point is searched in a certain number of the 32 regions determined by MV distribution prediction. The search points of the modified uneven multihexagon-grid search pattern are compressed to 1/5 (in Layer3-Range1) to 1/10 (in Layer1-Range1, 2, 3, 4) compared to the original pattern.

Moreover, in order to speed up the motion estimation, during the search process, the search patterns will be skipped as soon as the MV distribution prediction is equal to 0. In this case the MV is regarded as in center. This search strategy complies with the requirement of characteristic (2) mentioned in Section 3. A large number of experiments prove that the modified patterns and the skip mode can not only maintain motion estimation accuracy, but also avoid unnecessary search points effectively; thus, they are able to decrease the motion estimation encoding time.

### 4.2. Predict MV Distribution

To predict MV distribution, it is essential to calculate the size and the direction of the MV.

#### 4.2.1. Predict Size of MV

In this paper, the size of MV is obtained by comparing the current macroblock MV to the predicted MV threshold [24]. Some parameters which are related to the predicted MV threshold are defined as follows: (1 + *γ*)  pred_mincost_ represents the upper limit threshold of the MV prediction; (1 + *δ*)  pred_mincost_ represents the lower limit threshold of the MV prediction; in addition pred_mincost_ represents the minimum RD_mincost_ of the predicted MV. RD_mincost_ represents the rate-distortion value calculated during the motion estimation and it is defined as follows:

(3) Jmotion(mv,ref ∣ λmotion) =SAD[s,r(ref,m)]  +λmotion[R(m−pred)+R(ref)].

In motion estimation, *J*_motion_ is the RD_mincost_; pred is the predicted MV; *R* is the bit number of MV difference cost; *λ*_motion_ is the Lagrange parameter; *s*, *m*, ref, and SAD are mentioned in (1).

According to Figure 4, the size of MV will determine the predicted MV distributing in Layer1, Layer2, or Layer3. Define the following:

(4) $$\begin{array}{c} \text{R}{\text{D}}_{\text{mincost}}\leq \left(1+\gamma \right)\text{pre}{\text{d}}_{\text{mincost}},\\ \left(1+\gamma \right)\text{pre}{\text{d}}_{\text{mincost}}<\text{R}{\text{D}}_{\text{mincost}}<\left(1+\delta \right)\text{pre}{\text{d}}_{\text{mincost}},\\ \text{R}{\text{D}}_{\text{mincost}}\leq \left(1+\delta \right)\text{pre}{\text{d}}_{\text{mincost}} \end{array}$$

pred_mincost_ is obtained as the same method as the initial search point and it will state in the Section 4.3. In addiction *γ* and *δ* are defined as follows:

(5) γ=Bsize[blocktype]predmincost2−α1[blocktype]δ=Bsize[blocktype]predmincost2−α2[blocktype]α1[blocktype] =[−0.23,−0.23,−0.23,−0.25,−0.27,−0.27,−0.28]α2[blocktype] =[−2.39,−2.40,−2.40,−2.41,−2.45,−2.45,−2.48].

Bsize[blocktype] is the size of the current macroblock; blocktype is the serial number corresponding to the 7 size blocks, including 16 × 16, 8 × 16, 16 × 8, 8 × 8, 8 × 4, 4 × 8, and 4 × 4. *α*_1_[blocktype] and *α*_2_[blocktype] are a certain of parameters which are obtained by an enormous amount of experiments and the 7 values are connected with the 7 size blocks.

According to (4), the MV size prediction can be inferred by the rules as follows: the size of the current MV is equal to or less than the lower limit threshold, the motion activity belongs to low motion, and the MV is distributed in Origin or Layer1; the size of the current MV is between the upper limit threshold and the lower limit threshold, the motion activity belongs to middle motion, and the MV is distributed in Layer1 and Layer2; the size of the current MV is equal to or greater than the upper limit threshold, the motion activity belongs to high motion, and the MV is distributed in Layer3. In particular the method of predicting MV size is also used in other search patterns during the whole motion estimation.

#### 4.2.2. Predict Direction of MV

**Figure 7:**
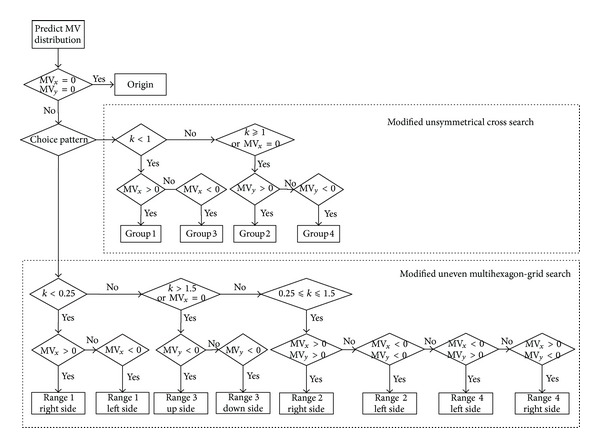
Flow chart of predicting the direction of MV.

Assuming that the coordinate of the MV is (MV_*x*_, MV_*y*_), and then the direction of MV can be described by Direction Vector, namely, $\vec{\text{MV}}=({\text{MV}}_{x},\text{M}{\text{V}}_{y})$. According to Figures 5 and 6, the direction of MV will determine the predicted MV distributing in Origin, Range1, Range2, Range3, Range4, Group1, Group2, Group3, and Group4. Define the parameter *k* which is used to represent the direction of MV as in (6). The corresponding search positions of $\vec{\text{MV}}$ is determined by *k* as shown in Figure 7:

(6) $$\begin{array}{c} k=\left|\frac{{\text{MV}}_{y}}{{\text{MV}}_{x}}\right|. \end{array}$$

Predicting the size and the direction of the MV can obtain MV distribution accurately. The MV distribution prediction will be the condition that the modified patterns select the search areas adaptively.

### 4.3. Framework of Proposed Scheme

Flow chart of the proposed motion estimation algorithm is as shown in Figure 12.

**Figure 8:**
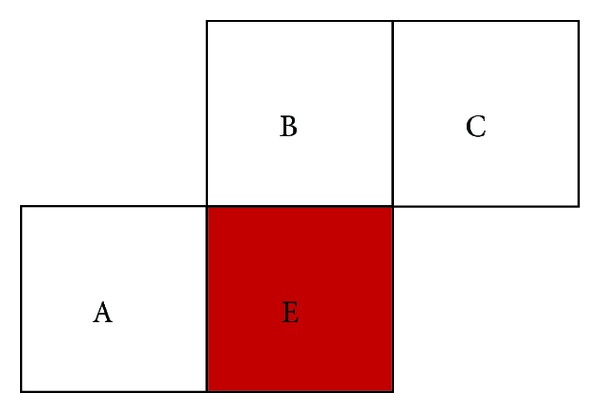
Median prediction.

**Figure 9:**
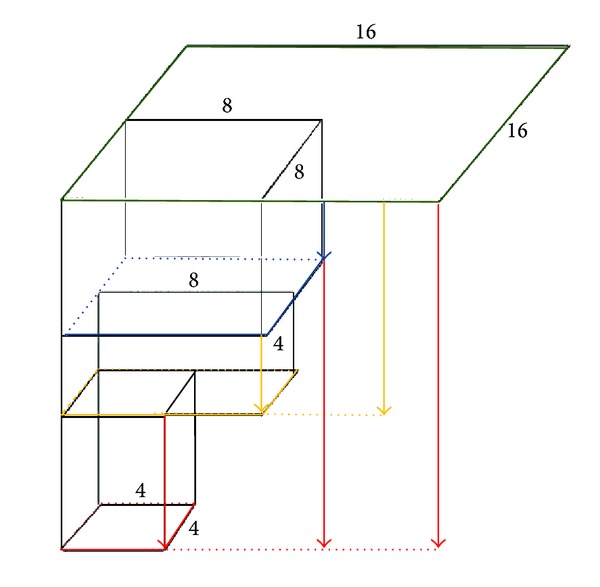
Up-layer prediction.

**Figure 10:**
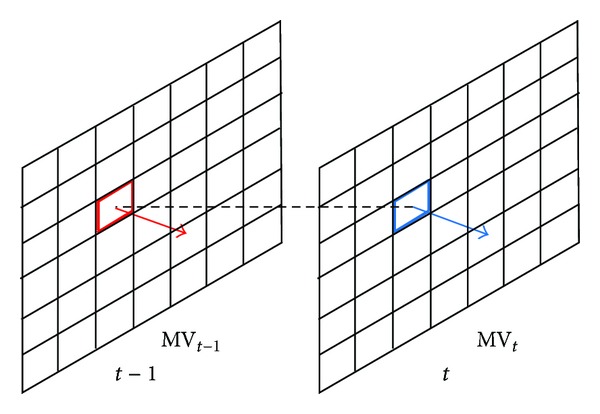
Corresponding-block prediction.

**Figure 11:**
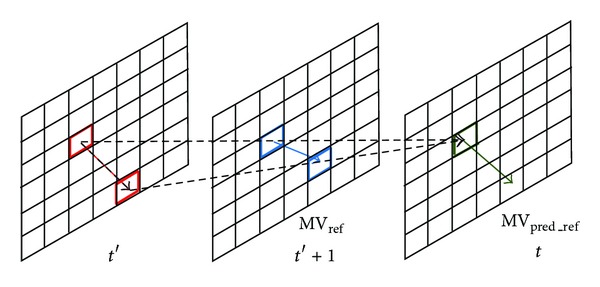
Neighboring reference frame prediction.

**Figure 12:**
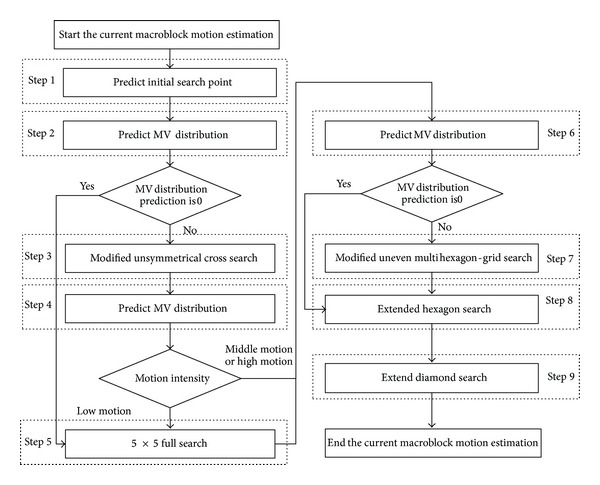
Flow chart of the proposed algorithm.

In Step 1 four prediction methods are used as shown in Figures 8, 9, 10, and 11, including the Median Prediction, the Up-Layer Prediction, the Corresponding-block Prediction, and the Neighboring Reference frame Prediction. Choose a MV which has the smallest rate-distortion cost as the searching center for the next step.

Median Prediction belongs to spatial prediction. It makes use of the relationship of the neighbor macroblocks in the same frame. Macroblock E is surrounded by the encoded Macroblock A, macroblock B, and macroblock C which have the same motion feature, so the current predicted MV_*E*_ is predicted by MV_*A*_, MV_*B*_, and MV_*C*_. The equation of the Median Prediction is described as follows:

(7) $$\begin{array}{c} {\text{MV}}_{E}=\text{median}\left[{\text{MV}}_{A},{\text{MV}}_{B},{\text{MV}}_{C}\right]. \end{array}$$

Up-Layer Prediction belongs to spatial prediction as well. It makes use of the various sizes of the macroblocks (16 × 16, 8 × 16, 16 × 8, 8 × 8, 8 × 4, 4 × 8, and 4 × 4). The small size macroblock can be predicted by the big size macroblock, because the small size macroblock can be regarded as segment of the big size macroblock. The equation of the Up-Layer Prediction is described as follows:

(8) $$\begin{array}{c} \text{M}{\text{V}}_{\text{current}}=\text{M}{\text{V}}_{\text{up\_layer}}. \end{array}$$

Corresponding-block Prediction belongs to temporal prediction. It makes use of the correlation of the corresponding frame. The different macroblocks with the same position in the current frame and the previous frame are likely to be part of a complete action, so the current predicted MV_*t*_ is predicted by MV_*t*−1_ with the same position in the previous frame. The equation of the Corresponding-block Prediction is described as follows:

(9) $$\begin{array}{c} \text{M}{\text{V}}_{t}=\text{M}{\text{V}}_{t-1}. \end{array}$$

Neighboring Reference frame Prediction belongs to temporal prediction as well. It makes use of the reference frames. Neighboring Reference frame Prediction is as the same as the Corresponding-block Prediction, but the former will take a quantity of neighbor frames to make the prediction more accurate. The current predicted MV_pred_ref_ is predicted by MV_ref_. The equation of the Neighboring Reference frame Prediction is described as follows:

(10) $$\begin{array}{c} \text{M}{\text{V}}_{\text{pred\_ref}}=\text{M}{\text{V}}_{\text{ref}}\times \frac{t-t^{\prime }}{t-t^{\prime }-1}. \end{array}$$

Regard the initial search point as the search center and perform the first time MV distribution prediction in Step 2. In this step the size and the direction of MV will be predicted. If the size of MV distribution prediction is 0, the Step 3 and Step 4 should be skipped, or the searching goes into next step.

In Step 3 choose the search Group in the modified unsymmetrical cross search pattern according to the direction of MV distribution prediction.

After finishing the modified unsymmetrical cross search, the second time MV distribution prediction is going to perform. In Step 4, the size of MV is predicted. When the MV is low motion, the 5 × 5 full search has to be performed. In this situation the MV is distributed in a low range around the center and the elaborate full search must be selected to ensure finding the most accurate matched point. The optional 5 × 5 full search in Step 5 not only devotes to searching the best matched point accurately but also avoids wasting search points effectively.

Then perform the third time MV distribution prediction in Step 6. In this step, the size and the direction of MV will be predicted. If the size of MV distribution prediction is 0, the Step 7 should be skipped, or choose the specific Layer and Range in modified uneven multihexagon-grid search.

In Steps 8 and 9 the extended hexagon search and the extend diamond search are performed. The extended hexagon search uses the hexagon pattern to search repeatedly until it obtains the best matched point. The extended diamond search uses the small diamond pattern to search repeatedly until it obtains the final MV. Then, the motion estimation is finished.

## 5. Performance Evaluations

### 5.1. Experimental Settings

To further test the effectiveness of the proposed algorithm, simulations have been performed over different intensity QCIF format (176 × 144) standard video sequences: CREW, HARBOUR, ICE, MOBILE, CITY, and COASTGUARD. Simulations present the results of comparing the proposed algorithm with UMHexagonS algorithm and NUMHexagonS algorithm by Y-PSNR, Bit-rate, ME-time, and average ME search points. Y-PSNR is the peak signal to noise ratio of luminance which is defined as follows:

(11) $$\begin{array}{c} \text{PSNR}=10\text{lo}{\text{g}}_{10}\left[\frac{255^{2}}{\text{MSE}\left(\text{MV}\right)}\right]; \end{array}$$

(12) $$\begin{array}{c} \text{MSE}\left(\text{MV}\right)=\frac{1}{MN}\overset{M}{\underset{x=1}{\sum }}\;\overset{N}{\underset{y=1}{\sum }}{\left[s\left(x,y,\text{MV}\right)-c\left(x,y\right)\right]}^{2}. \end{array}$$

In addiction, *s*(*x*, *y*, MV) is the current original image and *c*(*x*, *y*) is the compensated prediction image.

In order to compare the difference between the proposed algorithm, UMHexagonS algorithm, and NUMHexagonS algorithm, ME time Gain, Y PSNR Gain and Bits rate Gain are defined as follows:

(13) ME time Gain =[ME timeproposed−ME timeUMHexagonS/NUMHexagonS]ME timeUMHexagonS/NUMHexagonS  ×100%,

(14) Y PSNR Gain =[Y PSNRproposed−Y PSNRUMHexagonS/NUMHexagonS]Y PSNRUMHexagonS/NUMHexagonS  ×100%,

(15) Bits rate Gain =[Bits rateproposed−Bits rateUMHexagonS/NUMHexagonS]Bits rateUMHexagonS/NUMHexagonS  ×100%.

The reference software version is JM 18.4. The experimental conditions are shown in Table 2. In order to contain the completeness of the object movements, we set the previous 100 frames to be encoded, and other parameters are set by high profile. The representative frames of the standard sequences are as shown in Table 3. CREW is a high movement test sequence which includes lots of vertical movements and the foreground is the main movements; HARBOUR is a low movement test sequence; the movement of ICE is the highest movements of the six sequences and its foreground has quiet high motion; MOBLE and COASTGUARD have a large numbers of horizontal movements; CITY is a revolving movement test sequence including its foreground and background.

### 5.2. Experimental Results

To make sure the experimental results meaningful, all the standard sequences are generated in the same configuration. The experimental results are shown in Table 4. Compared to UMHexagonS algorithm, the proposed algorithm maintains a good quality of the reconstruction video, PSNR falls by barely 0.04% on average and Bit rate increases by mere 0.58% on average. Compared to NUMHexagonS algorithm, PSNR falls by barely 0.02% on average; Bit rate increases by mere 0.48% on average. The proposed algorithm keeps H.264/AVC high compression ratio performance. It can reduce motion estimation time up to 20.86% on average compared to UMHexagonS algorithm and reduce motion estimation time up to 13.24% on average compared to NUMHexagonS algorithm as well. The experimental results verify the rationality of the novel design including the modified patterns and the search point distribution. Hence, the search strategy based on MV distribution prediction is effective.

### 5.3. Rate-Distortion Performance Evaluation

**Figure 13:**
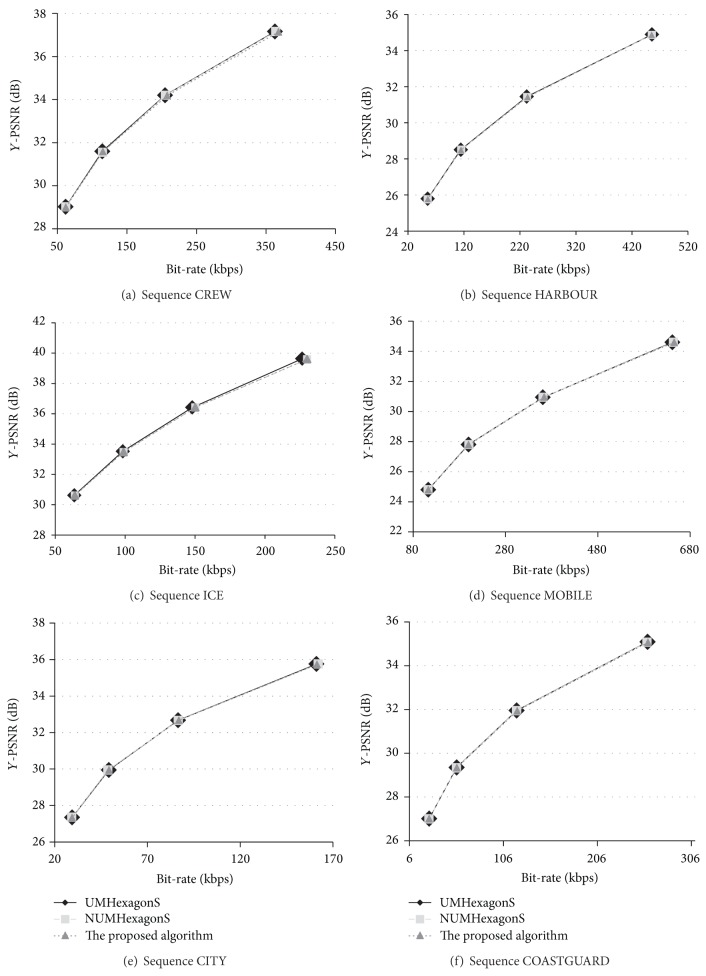
Comparison of rate-distortion performance between UMHexagonS, NUMHexagonS, and the proposed algorithm.

In Figure 13 the rate-distortion curves are drawn according to Table 4 under various QPs. Comparison of rate-distortion performance between UMHexagonS, NUMHexagonS, and the proposed algorithm, the three curves are vary closed, which means that the proposed algorithm has an advantage to maintain low compression bit rate and high reconstruction quality of H.264/AVC. HARBOUR and ICE are as the typical video sequences to represent low motion and high motion, respectively. Rate-distortion performance of the sequence HARBOUR shows that the proposed algorithm fits to low motion video with a good performance. The quality of high motion sequence ICE decreases a little under low QPs, and under high QPs the proposed algorithm performs the same good as the low motion videos. Thus, the proposed algorithm has a negligible loss of quality and Bit rate, while completing the novel motion estimation scheme.

### 5.4. ME Search Points Performance Evaluation

**Figure 14:**
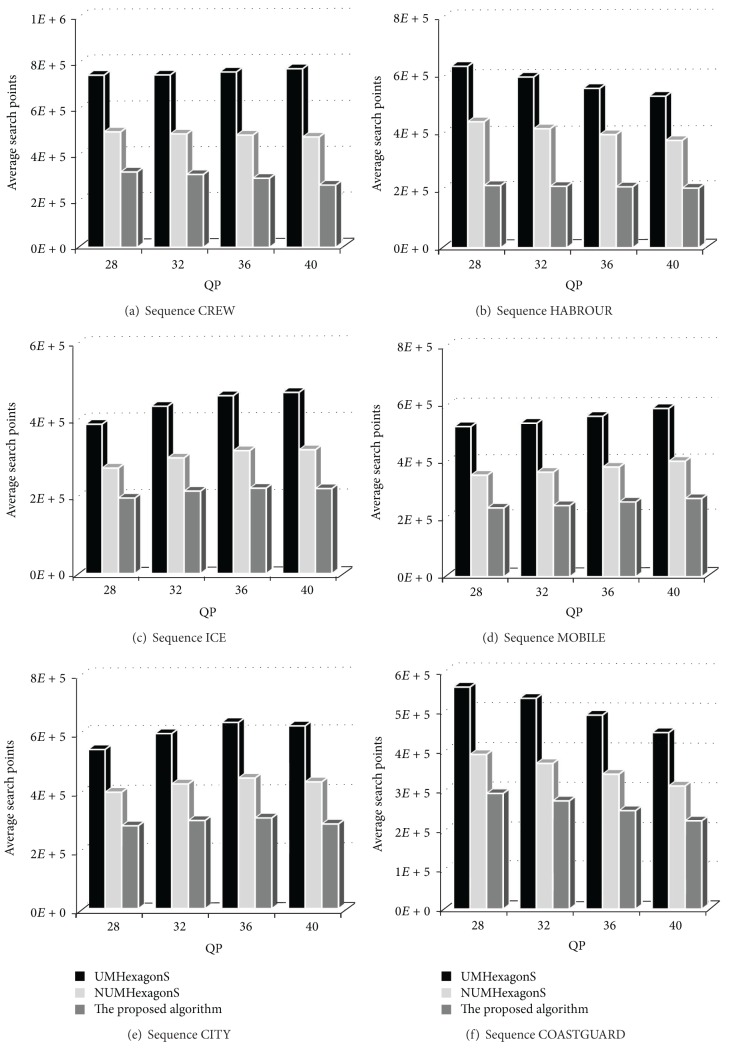
Comparison of average ME search points between UMHexagonS, NUMHexagonS, and the proposed algorithm.

To compare the efficiency between UMHexagonS algorithm, NUMHexagonS algorithm, and the proposed algorithm accurately, the search points are counted during ME process and drawn in Figure 14. Search points histograms show that the proposed algorithm reduces up to 54.62% and 36.88% search points, respectively, on average compared to UMHexagonS algorithm and NUMHexagonS algorithm, which improves motion estimation real-time performance considerably. The proposed algorithm has an obvious and stabile optimization and reduces the complexity of motion estimation algorithm architecture effectively.

Because each of the 7 macroblock mode should be performed the motion estimation to get the MV for mode decision, the proposed algorithm can save several-fold encoding time by reducing the search points in each time motion estimation. The MV distribution prediction is performed three times to locate the MV before patterns search which is the key point to speed up the motion estimation meanwhile maintaining the search accuracy.

## 6. Conclusion

In this paper, a flexible and fast ME scheme is proposed. It makes full use of the motion continuity and motion integrity of the video coding objects and combined MV distribution prediction with the new search patterns to make the search position more accurate. The proposed MV distribution prediction algorithm chooses search direction by predicting the MV direction and selects search level by predicting the MV size. Then, the proposed adaptive ME scheme further narrowed the search range of ME and reduces the unnecessary search points. The experimental results show that the proposed algorithm decreased by 20.86% of ME time and 54.62% of ME search points, respectively, compared to UMHexagonS algorithm (JM18.4), while maintaining the quality of the original structure and reconstruction bit streams. The proposed algorithm improves the performance of H.264/AVC real-time encoding effectively, and it can be combined with other fast video coding techniques such as fast mode prediction algorithm and reference frame selection algorithm to improve the encoding speed. The proposed prediction concept for ME estimation in this paper can be further used in HEVC which is the next phase of research direction.

## Figures and Tables

**Table 1:** MV distribution probabilities of different video sequences.

Region of MV distribution	Video sequence name						
	FOOTBALL	BUS	CITY	CREW	HARBOUR	ICE	MOBILE
Origin (%)	16.84	6.11	6.01	13.67	42.90	20.58	1.97
Layer1-Range1 (%)	5.09	36.18	29.34	8.81	16.51	13.64	39.38
Layer1-Range2 (%)	3.82	7.17	13.74	5.18	2.27	5.21	10.04
Layer1-Range3 (%)	9.36	13.74	26.86	23.47	21.78	10.56	18.02
Layer1-Range4 (%)	1.09	3.49	6.84	2.89	0.66	2.38	5.23
Layer2-Range1 (%)	2.08	9.49	3.43	2.56	3.99	4.42	9.68
Layer2-Range2 (%)	1.24	1.90	1.72	1.39	0.56	1.76	2.58
Layer2-Range3 (%)	2.78	3.14	3.6	5.76	5.71	3.92	4.22
Layer2-Range4 (%)	0.45	0.93	0.85	0.77	0.18	0.86	1.24
Layer3-Range1 (%)	18.44	10.31	2.88	9.27	2.18	14.97	4.40
Layer3-Range2 (%)	8.61	1.91	1.07	3.94	0.23	4.95	1.05
Layer3-Range3 (%)	25.96	4.72	3.16	20.13	2.95	14.45	1.78
Layer3-Range4 (%)	4.23	0.91	0.52	2.15	0.08	2.30	0.42

**Table 2:** Experimental configuration.

PC configuration	
CPU	3.4 GH
RAM	4 GB
Operating system	Windows 7

Encode configuration	

Frames to be encoded	100
Frame rate	30
GOP structure	IPPP
Quantization parameter	28, 32, 36, 40
Numbers of reference frames	5
Search range	32
Entropy coding	CABAC
Rate distortion optimization	Enabled

**Table 3:** Standard video sequences.

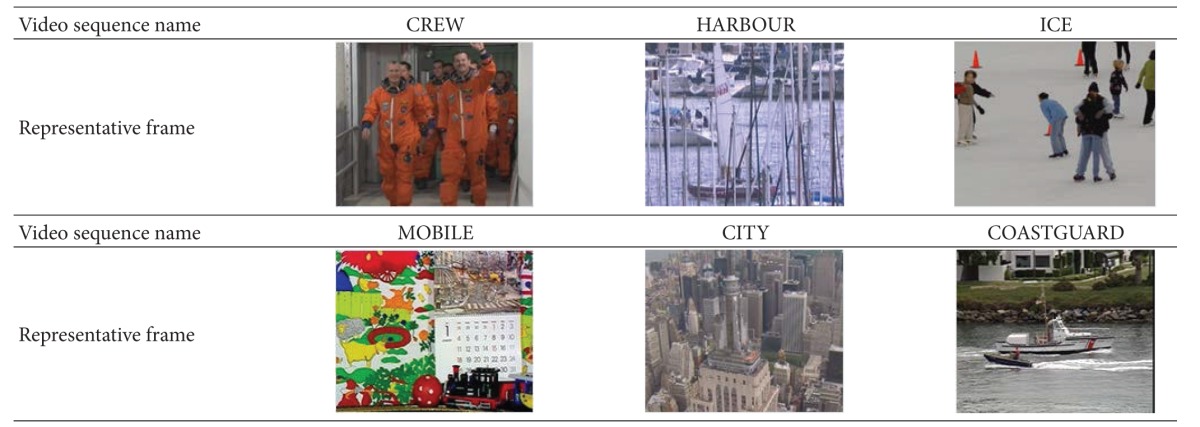

**Table 4:** Performance of the proposed algorithm compared.

Video sequence name	QP	The proposed algorithm compared to UMHexagonS			The proposed algorithm compared to NUMHexagonS		
		ME time Gain (%)	Y PSNR Gain (%)	Bits rate Gain (%)	ME time Gain (%)	Y PSNR Gain (%)	Bits rate Gain (%)
CREW	28	−25.85	−0.05	1.39	−14.65	−0.04	1.08
	32	−25.83	−0.03	1.53	−14.37	−0.02	1.28
	36	−26.76	−0.04	0.71	−15.04	0.11	0.38
	40	−28.52	−0.06	0.50	−16.81	−0.08	−0.08

HARBOUR	28	−26.24	−0.01	−0.10	−17.50	0.03	−0.05
	32	−24.60	−0.05	0.65	−16.53	0.03	0.40
	36	−22.26	−0.03	0.51	−15.17	−0.01	−0.08
	40	−21.20	−0.03	0.29	−14.33	0.09	0.63

ICE	28	−16.41	−0.10	1.54	−9.12	−0.03	0.98
	32	−17.52	0.01	1.51	−9.55	−0.09	1.14
	36	−18.15	−0.14	0.59	−10.08	0.06	0.88
	40	−17.89	−0.05	0.60	−8.91	−0.29	0.34

MOBILE	28	−21.23	−0.01	0.61	−11.78	−0.01	0.28
	32	−21.00	0.05	0.84	−11.62	0.03	0.78
	36	−21.30	−0.09	0.12	−11.92	−0.08	−0.37
	40	−21.65	−0.04	0.29	−12.25	0.00	0.43

CITY	28	−15.97	−0.05	0.17	−9.51	−0.05	−0.39
	32	−17.60	0.02	0.53	−10.11	0.05	0.37
	36	−18.64	0.01	0.16	−10.73	0.00	1.21
	40	−19.28	−0.04	0.07	−11.72	−0.06	0.58

COASTGUARD	28	−18.43	−0.08	−0.08	−11.55	−0.03	0.40
	32	−18.10	−0.04	−0.04	−18.10	−0.04	0.55
	36	−18.76	−0.04	−0.04	−18.76	−0.04	0.53
	40	−17.54	0.00	0.00	−17.54	0.00	0.15
